# Health-related quality of life and tuberculosis: a longitudinal cohort study

**DOI:** 10.1186/s12955-015-0250-4

**Published:** 2015-05-27

**Authors:** Melissa Bauer, Sara Ahmed, Andrea Benedetti, Christina Greenaway, Marek Lalli, Allison Leavens, Dick Menzies, Claudia Vadeboncoeur, Bilkis Vissandjée, Ashley Wynne, Kevin Schwartzman

**Affiliations:** Respiratory Epidemiology and Clinical Research Unit, McGill University, Montreal, QC Canada; Department of Epidemiology, Biostatistics and Occupational Health, McGill University, Montreal, QC Canada; School of Physical and Occupational Therapy, McGill University, Montreal, QC Canada; Division of Infectious Diseases and Clinical Epidemiology, Sir Mortimer B. Davis Jewish General Hospital, Montreal, QC Canada; Faculté des sciences infirmières – School of Nursing, Université de Montréal, Montreal, QC Canada

**Keywords:** Tuberculosis, Health-related quality of life, SF-36, Linear mixed model regression

## Abstract

**Background:**

Active tuberculosis (TB) disease can impose substantial morbidity, while treatment for latent TB infection (LTBI) has frequent side effects. We compared health-related quality of life (HRQOL) between persons diagnosed and treated for TB disease, persons treated for LTBI, and persons screened but not treated for TB disease or LTBI, over one year following diagnosis/initial assessment.

**Methods:**

Participants were recruited at two hospitals in Montreal (2008–2011), and completed the Short Form-36 version 2 (SF-36) at baseline, and at 1, 2, 4, 6, 9, and 12 months thereafter. Eight domain scores and physical and mental component summary (PCS and MCS, respectively) scores were calculated from responses. Linear mixed models were used to compare mean scores at each evaluation and changes in scores over consecutive evaluations, among participants treated for TB disease and those treated for LTBI, each compared to the control group.

**Results:**

Of the 263 participants, 48 were treated for TB disease, 105 for LTBI, and 110 were control participants. Fifty-four percent were women, mean age was 35 years, and 90% were foreign-born. Participants treated for TB disease reported significantly worse mean scores at baseline compared to control participants (mean PCS scores: 50.0 vs. 50.7; mean MCS scores: 46.4 vs. 51.1), with improvement in mean MCS scores throughout the study period. Scores reported by participants treated for LTBI and control participants were comparable throughout the study.

**Conclusion:**

TB disease is associated with decrements in HRQOL as measured by the SF-36. This is most pronounced during the weeks after diagnosis and treatment initiation, but is no longer evident after two months.

**Electronic supplementary material:**

The online version of this article (doi:10.1186/s12955-015-0250-4) contains supplementary material, which is available to authorized users.

## Background

Individuals diagnosed with tuberculosis (TB) disease report notable decrements in health-related quality of life (HRQOL), in relation to both physical and psychological well-being [1,2]. Treatment typically requires a combination of 4 drugs in the initial phase, with a minimum treatment period of 6 months [3]. Treatment guidelines recommend a patient-centered approach to address both medical and psychological needs of patients with TB disease [4].

In high-income countries, like Canada, many patients diagnosed with latent TB infection (LTBI) receive treatment. This treatment usually involves isoniazid (INH) taken daily for 9 months; treatment completion is often hampered by drug intolerance and inconvenient treatment and clinic visit schedules [5,6].

An overwhelming majority of individuals screened and treated for LTBI and TB disease in high-income countries are recent immigrants [3,7,8]. A key challenge of research describing HRQOL of patients treated for LTBI and TB disease is to tease apart the impact of diagnosis and treatment from that of typical stressors facing the immigrant population such as language barriers, social isolation, unemployment, unstable housing, access to health care, *etc*. [9,10].

Our study reports longitudinal measures of HRQOL over the year following diagnosis, as measured with the Short Form-36 version 2 (SF-36) questionnaire, reported by patients recruited from two hospitals in Montreal, Canada who were treated for TB disease or LTBI [11,12]. To distinguish the impact of treatment for TB disease and LTBI from other stressors related to immigration (*e.g.* socioeconomic instability and unemployment), we also include concurrent measures of HRQOL reported by individuals screened for TB but found not to require treatment. This participant group remained untreated throughout the study period.

Our aim was to measure HRQOL of patients treated for TB disease and LTBI at each milestone of treatment, along with untreated individuals with a similar socio-demographic profile to treated participants. These measures may assist health care professionals understand the health impact of TB disease, LTBI, and their treatment, and may inform targeted interventions to improve patients’ health and well-being.

## Methods

### Study population and participant recruitment

The study population, sample recruitment, and study design have been detailed elsewhere [13]. Briefly, study participants were recruited between June, 2008 and October, 2011 at two hospitals in Montreal - the Montreal Chest Institute (MCI) and the Jewish General Hospital (JGH). Reason for hospital referral of potential participants was captured at the initial evaluation.

The group of participants diagnosed with TB disease included patients initially hospitalized and those treated solely as outpatients; all participants treated for TB disease had culture-confirmed disease. Participants treated for LTBI were diagnosed with asymptomatic infection, typically based on positive results from a tuberculin skin test (TST) and/or an Interferon-γ Release Assay (IGRA), and/or presence of scarring on a chest radiograph. Participants in the control group were evaluated for possible TB disease and/or LTBI, and judged not to require treatment of any kind. All participants were recruited to the study within two weeks of TB screening/treatment initiation.

Individuals with multi-drug resistant TB disease, or concomitant physical or mental illness likely to affect their HRQOL were excluded from the study. Furthermore, all participants were required to understand English or French and to be at least 18 years of age at study enrollment.

Participants’ written informed consent was obtained before the initial interview. Research ethics committee approval was obtained from both the MCI and JGH. The study was performed in accordance with the ethical standards established in the 1964 Declaration of Helsinki and its later amendments.

### Study design

Participants were evaluated during clinic or home visits at 1, 2, 4, 6, 9, and 12 months post-baseline, corresponding to important milestones in TB treatment regimens [4]. We used frequency matching to balance the proportions of immigrants across the three participant groups; for every ten participants recruited to the group treated for TB disease, the proportions of immigrants recruited into the LTBI and control groups were equalized to the proportion in the TB disease group.

### Study measurements

At the baseline interview, participants were evaluated for language ability and completed questionnaires describing their socio-demographic and clinical characteristics. Included in the socio-demographic questionnaire was a question about immigration status in Canada - Canadian citizen/permanent resident, immigrant applicant, accepted refugee, refugee claimant, temporary resident (with an employment visa), or other category (student or visitor visa).

Clinical information (*e.g.* diagnostic test results, prescribed treatment regimens, other concomitant conditions and/or medications) was verified in participants’ medical charts at each interview. Participant-reported changes to medications and adverse events were recorded at all follow-up visits and verified in participants’ medical charts.

Participants also completed the written SF-36 questionnaire, version 2 in Canadian English or Canadian French at baseline and at all follow-up evaluations [12]. The SF-36 written questionnaire contains 36 questions and generates scores for 8 domains of HRQOL - physical functioning, role physical, bodily pain, general health, vitality, social functioning, role emotional, and mental health. Physical and mental component summary (PCS and MCS, respectively) scores can also be derived from responses [12].

At each interview, the research assistant scanned the questionnaires for any unanswered questions and asked the participant to provide missing responses, unless the question was intentionally left unmarked by the participant.

We conducted double data entry and resolved discrepancies against paper source documents and by consensus discussion where appropriate.

### Main statistical analyses

Socio-demographic characteristics and clinical features were summarized using descriptive statistics for each participant group at each assessment. Distributions of SF-36 domain scores and component summary scores using an oblique factor solution were summarized for each participant group at each evaluation [14].

The associations of PCS and MCS scores with participant group (TB disease, LTBI, control) were the primary focus of this analysis. Characteristics considered *a priori* confounders of these associations were examined quantitatively [15-18]. An *a priori* level of statistical significance was set at α = 0.05.

In addition, an *ad hoc* analysis was performed stratifying participants treated for TB disease into severe cases (those hospitalized for treatment of TB disease at baseline or acid fast smear-positive at baseline) and non-severe cases (those never hospitalized for TB treatment and smear-negative at baseline).

Crude effect sizes, examining change in scores over time within each participant group, were calculated at each assessment and evaluated using Cohen’s criteria; a statistically meaningful change in mean scores was defined *a priori* as an effect size ≥ 0.50 [19].

A minimal clinically important difference (MCID), or the smallest difference in a domain score which patients perceive an improvement or a worsening, is unknown in the TB context [20]. MCID thresholds for SF-36 domain scores published for patients with other respiratory diseases were compared to our results [21].

Multivariable linear mixed models were used to compare each PCS and MCS score reported by each of the treated participant groups to the control group, with the control group as the referent for all models. A time*participant group interaction term was included to account for different score patterns over time between the participant groups. Random intercepts, random slopes, and spatial covariance structures using visit number were incorporated into the models. Age at baseline, sex, and additional factors determined to be important confounders were included as covariates in all adjusted models. Model fit was assessed using the Akaike Information Criterion (AIC) [22].

Adjusted estimates of mean PCS and MCS scores for each participant group at each assessment were calculated from final model estimates. Changes in adjusted scores between successive interviews and from baseline were also calculated from these estimates. Parametric 95% confidence intervals (CI) were calculated for adjusted estimates of mean scores and changes in mean scores [22].

Sample size calculations were based on the primary objective of the cohort study – to derive health utility scores over the year following diagnosis for the three participant groups [13]. Sample size calculations indicated that 40 participants treated for TB disease should be recruited to the study to detect a change in health utility scores evaluated with the Standard Gamble of 0.003/month over 12 months with 80% power, ρ = 0.8, and α = 0.05 [13]. Additional information about health utility scores measured in this cohort is available in reference [13].

### Sensitivity analyses

Multiple imputation (MI) was used to quantify the impact of missing data on the final model estimates [23].

Profiles potentially differed between individuals who (1) agreed to participate vs. those who refused participation, and (2) those who attended follow-up visits, vs. those who missed a given visit but returned for later visits, vs. those permanently lost to follow-up. Age at recruitment/initial interview and sex were available for all individuals approached by the research assistant. Individuals in each diagnosis group were stratified by sex and reason for refusing study participation; mean ages were calculated for each stratum. Among study participants, we calculated mean component summary scores according to participant group, visit number, and attendance status (attended, missed, or lost to follow-up) at the next scheduled visit.

All statistical analyses were conducted using SAS statistical software (version 9.3; SAS Institute, Cary, North Carolina, United States); graphs were created using Microsoft Excel (2010; Redmond, Washington, United States) [24,25].

## Results

### Study sample

**Figure 1:**
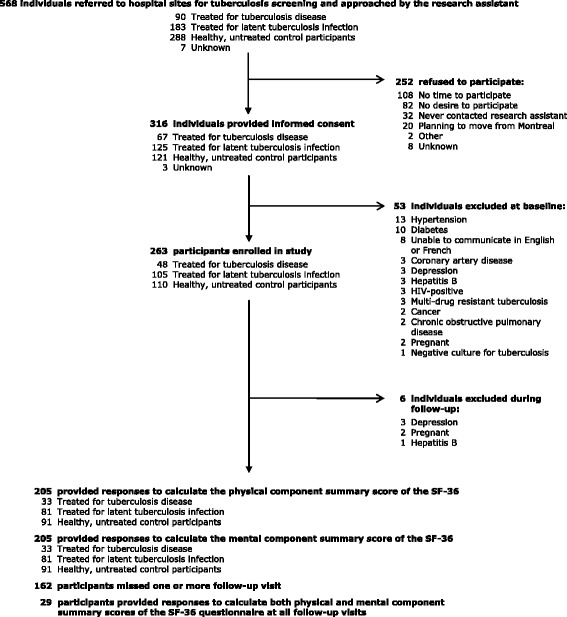
Participant selection in longitudinal study with recruitment from June 2008 – October 2011.

Of the 568 individuals referred to either the MCI or the JGH for TB assessment and approached by the research assistant, 53 (17%) were ineligible for participation at baseline; of the remainder, 252 refused to participate. The refusal rate was higher among men (60%) than women (40%), and among those found not to require treatment for TB disease or LTBI (67%) compared to those diagnosed with TB disease (9%) or LTBI (23%). In total, 263 participants were enrolled in the study - 48 were treated for TB disease, 105 were treated for LTBI, and 110 were control participants. During follow-up, an additional 3 participants were determined ineligible for further participation and were excluded from subsequent assessments [Figure 1].

Among participants treated for TB disease, 21 (44%) missed at least one follow-up visit, and 4 (8%) participants provided data to permit calculation of both PCS and MCS scores at every follow-up visit. Seventy-six (72%) participants treated for LTBI and 65 (59%) participants in the control group missed at least one follow-up visit. We were able to calculate both PCS and MCS scores at all visits for 7 (7%) participants treated for LTBI and 18 (16%) participants in the control group [Figure 1].

### Participant characteristics

Table 1 describes key characteristics reported by participants at baseline. Fifty-four percent of participants were women, and 90% of participants were foreign-born; more than half were Canadian citizens or permanent residents.

**Table 1:** Participant characteristics at the initial interview by participant group and sex (N = 263)

**Characteristics**	**Treated for tuberculosis disease**			**Treated for latent tuberculosis infection**			**Screened for tuberculosis but healthy and untreated**		
	**Total (%)**	**Men (%)**	**Women (%)**	**Total (%)**	**Men (%)**	**Women (%)**	**Total (%)**	**Men (%)**	**Women (%)**
Sample size	48 (18)	27 (56)	21 (44)	105 (40)	45 (43)	60 (57)	110 (42)	50 (45)	60 (55)
Mean (standard deviation) age - years	37 (12)	38 (15)	36 (7)	33 (9)	34 (9)	33 (10)	35 (8)	34 (8)	35 (8)
Region of origin									
Africa	15 (31)	12 (44)	3 (13)	29 (28)	18 (40)	11 (19)	34 (31)	16 (32)	18 (30)
Asia	18 (38)	8 (29)	10 (47)	28 (27)	8 (18)	20 (33)	21 (19)	11 (22)	10 (17)
Eastern Europe	3 (6)	1 (4)	2 (10)	5 (5)	3 (7)	2 (3)	12 (11)	5 (10)	7 (12)
Western Europe	1 (2)	1 (4)	0 (0)	5 (5)	3 (7)	2 (3)	10 (9)	5 (10)	5 (8)
Central America	3 (6)	1 (4)	2 (10)	20 (18)	6 (13)	14 (24)	18 (16)	7 (14)	11 (18)
North America	5 (11)	3 (11)	2 (10)	11 (10)	5 (11)	6 (10)	11 (10)	4 (8)	7 (12)
South America	3 (6)	1 (4)	2 (10)	7 (7)	2 (4)	5 (8)	4 (4)	2 (4)	2 (3)
Median (inter-quartile range) duration in Canada (years)	2 (0–7)	2 (0–10)	2 (0–7)	3 (1–9)	1 (0–6)	5 (2–10)	2 (1–9)	2 (1–7)	2 (1–9)
Reason for hospital referral									
Pre-landing refugee or immigrant screening	9 (19)	5 (19)	4 (19)	6 (6)	3 (7)	3 (5)	17 (15)	10 (20)	7 (12)
Post-landing surveillance	7 (15)	3 (11)	4 (19)	11 (10)	7 (16)	4 (7)	7 (6)	4 (8)	3 (5)
Tuberculin Skin Test, not contact of patient with diagnosed TB disease	1 (2)	0 (0)	1 (5)	64 (61)	22 (49)	42 (70)	67 (61)	27 (54)	40 (67)
Contact of patient with diagnosed TB disease	1 (2)	0 (0)	1 (5)	18 (17)	10 (22)	8 (13)	5 (5)	3 (6)	2 (3)
Symptomatic of TB disease^‡^	24 (50)	17 (63)	7 (33)	0 (0)	0 (0)	0 (0)	0 (0)	0 (0)	0 (0)
Other	6 (13)	2 (7)	4 (19)	6 (6)	3 (7)	3 (5)	0 (0)	0 (0)	0 (0)
Unknown	0 (0)	0 (0)	0 (0)	0 (0)	0 (0)	0 (0)	14 (13)	6 (12)	8 (13)
Smoking status									
Ever cigarette smoker*	18 (38)	14 (52)	4 (19)	24 (23)	15 (33)	9 (15)	23 (21)	14 (28)	9 (15)
Current cigarette smoker^†^	11 (23)	9 (33)	2 (10)	8 (8)	5 (11)	3 (5)	10 (9)	5 (10)	5 (8)
Median (inter-quartile range) pack-years smoking	13 (6–19)	17 (8–20)	5 (3–9)	4 (1–16)	2 (1–16)	5 (2–12)	6 (3–18)	5 (1–18)	6 (4–21)

^‡^Common symptoms of TB disease include chronic cough of at least two weeks duration, fever, and night sweats. Other symptoms may include hemoptysis, anorexia, weight loss, and chest pain. *A participant who never smoked is someone who smoked less than 20 packs of cigarettes or 400 grams of tobacco in a lifetime or less than 1 cigarette a day for 1 year.

^†^A participant who is a current smoker smoked cigarettes as of 1 month before the initial interview.

Of the 263 participants, 50% were referred to a study site for evaluation of a positive TST result, but were not contacts of persons with TB disease. This group represented over 60% of the participants treated for LTBI, and of those in the control group. Fifty percent of the participants treated for TB disease were referred to a study site because of symptoms, and 19% and 15% were referred for TB screening and surveillance, respectively, in the context of recent immigration. Sixty-three percent of participants treated for TB disease missed some work or school during the study period due to their diagnosis and treatment [Table 1].

Clinical characteristics are reported in Additional file 1. Of participants with TB disease, 40 (83%) had pulmonary disease, of whom 13 (33%) had cavitary disease. Twenty (42%) received directly observed therapy, and 22 (46%) were hospitalized; median stay was 14 days (Inter-quartile range, (IQR) 11, 23).

Among participants treated for LTBI, 20 (19%) had abnormal chest radiographs. All participants treated for LTBI self-administered their medication [Additional file 1].

At the initial assessment, a greater proportion of participants treated for TB disease reported at least one other concomitant health condition (67%) or one other medication (60%) compared to participants treated for LTBI (30% and 10%, respectively) or control participants (12% and 5%, respectively) [Additional file 1].

Sixteen (34%) participants treated for TB disease and 20 (38%) participants treated for LTBI reported at least 1 episode of treatment intolerance between the baseline and 1-month evaluations. These numbers decreased from the 1-month through the 9-month visits. No participant experienced an adverse event that led to hospitalization.

All participants treated for TB disease completed treatment. Eighty of the 94 participants (85%) prescribed the 9-month regimen of INH for the treatment of LTBI completed their treatment, and 8 of the 10 participants (80%) prescribed the 4-month regimen of Rifampin for the treatment of LTBI completed their treatment.

### Findings from univariable analyses

**Figure 2:**
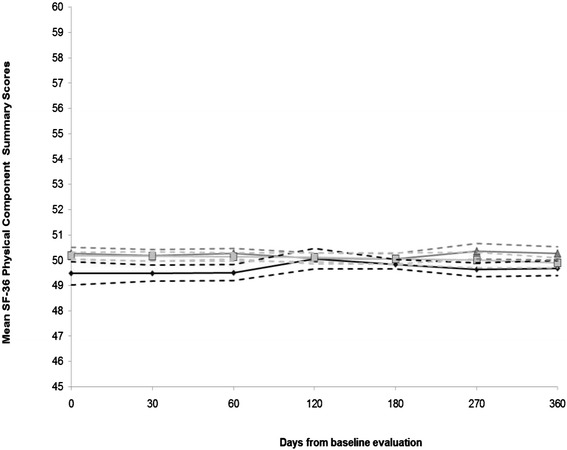
Mean physical component summary scores and 95% confidence intervals from each visit by participant group.

**Figure 3:**
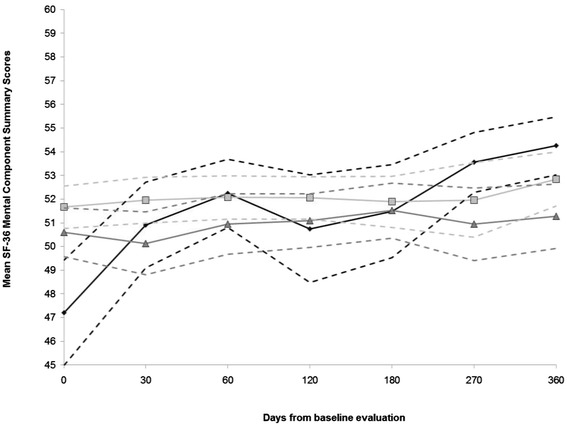
Mean mental component summary scores and 95% confidence intervals from each visit by participant group.

Participants treated for TB disease reported somewhat lower mean PCS scores during the first two months of treatment and significantly lower mean MCS scores at baseline, compared to other participant groups [Figures 2, 3]. Statistical improvement in mean PCS scores was observed from the 2- to the 4-month visits, as indicated by effect sizes ≥ 0.50 [19] [Table 2]. However, mean PCS scores declined and resembled the mean PCS score at baseline [Table 2, Figure 2]. Statistical improvements in mean MCS scores were observed from baseline to each follow-up visit [19] [Table 3].

**Table 2:** Mean physical component summary scores and effect sizes by participant group and sex (N = 205)

	**Participants**	**Treated for tuberculosis disease**				**Treated for latent tuberculosis infection**				**Screened for tuberculosis but healthy and untreated**			
**VISIT**	**BY SEX**	**N (%)**	**Mean score (Standard Deviation)**	**Effect size**_**1**_^**a**^	**Effect size**_**2**_^**b**^	**N (%)**	**Mean score (Standard Deviation)**	**Effect size**_**1**_^**a**^	**Effect size**_**2**_^**b**^	**N (%)**	**Mean score (Standard Deviation)**	**Effect size**_**1**_^**a**^	**Effect size**_**2**_^**b**^
Baseline	Total	33	49.5 (1.3)	-	-	81	50.3 (1.1)	-	-	91	50.2 (0.7)	-	-
	Men	19 (58)	49.6 (1.5)	-	-	37 (46)	50.2 (0.9)	-	-	41 (45)	50.1 (0.6)	-	-
	Women	14 (42)	49.4 (1.2)	-	-	44 (54)	50.4 (1.2)	-	-	50 (55)	50.3 (0.8)	-	-
1 month	Total	36	49.5 (1.0)	0.0	-	58	50.2 (0.9)	−0.1	-	75	50.2 (0.8)	0.0	-
	Men	21 (58)	49.6 (1.1)	0.0	-	22 (38)	50.2 (0.6)	0.1	-	38 (51)	50.2 (0.9)	0.2	-
	Women	15 (42)	49.4 (0.8)	0.0	-	36 (62)	50.2 (1.1)	−0.1	-	37 (49)	50.1 (0.8)	−0.2	-
2 months	Total	39	49.5 (1.0)	0.0	0.0	52	50.3 (0.8)	0.0	0.1	77	50.1 (0.8)	−0.1	0.0
	Men	23 (59)	49.7 (1.2)	0.1	0.1	23 (44)	50.2 (0.8)	0.0	0.0	37 (48)	50.1 (0.8)	0.1	0.0
	Women	16 (41)	49.2 (0.5)	−0.2	−0.2	29 (56)	50.3 (0.8)	0.0	0.1	40 (52)	50.1 (0.8)	−0.2	0.0
4 months	Total	32	50.1 (1.2)	**0.5**	**0.5**	53	50.1 (0.8)	−0.2	−0.2	56	50.1 (0.7)	−0.1	0.0
	Men	19 (59)	50.4 (1.4)	**0.6**	**0.5**	26 (49)	50.0 (0.7)	−0.2	−0.2	24 (75)	50.2 (0.6)	0.1	0.0
	Women	13 (41)	49.6 (0.4)	0.3	**0.8**	27 (51)	50.1 (0.8)	−0.2	−0.2	32 (25)	50.1 (0.7)	−0.2	−0.1
6 months	Total	33	49.8 (0.6)	0.4	−0.2	44	50.1 (0.7)	−0.2	0.0	55	50.1 (0.9)	−0.1	−0.1
	Men	18 (55)	50.0 (0.6)	0.4	−0.4	25 (57)	50.1 (0.5)	−0.1	0.1	27 (49)	50.1 (0.7)	0.0	−0.1
	Women	15 (45)	49.7 (0.5)	0.3	0.2	19 (43)	50.0 (0.9)	−0.4	−0.2	28 (51)	50. (1.0)	−0.2	0.0
9 months	Total	30	49.6 (0.8)	0.1	−0.3	37	50.4 (1.0)	0.1	0.3	44	50.0 (1.0)	−0.2	−0.1
	Men	18 (60)	49.5 (0.9)	0.0	**−0.5**	20 (54)	50.3 (0.8)	0.2	0.3	18 (44)	49.7 (0.8)	**−0.5**	−0.4
	Women	12 (40)	49.8 (0.5)	0.4	0.1	17 (46)	50.4 (1.1)	0.0	0.4	26 (59)	50.2 (1.1)	0.0	0.1
12 months	Total	21	49.7 (0.7)	0.2	0.1	42	50.3 (0.9)	0.0	−0.1	49	49.9 (0.7)	−0.4	−0.1
	Men	13 (62)	49.7 (0.8)	0.2	0.2	21 (50)	50.4 (0.9)	0.2	0.0	23 (47)	50.0 (0.6)	−0.2	0.3
	Women	8 (38)	49.6 (0.5)	0.2	−0.4	21 (50)	50.2 (0.9)	−0.2	−0.2	26 (53)	49.8 (0.8)	**−0.5**	−0.4

Effect size conveys the estimated magnitude in differences in two measures of scores. An effect size ≥0.50 indicates a statistically meaningful change in mean scores and is indicated in bold font in the table. 15

^a^Effect Size_1_ is the effect size of the change in mean physical component summary scores from the baseline visit to the given visit.

^b^Effect size_2_ is the effect size of the change in mean physical component summary scores from the previous visit to the given visit, e.g. effect size_2_ for the 4-month visit is the effect size of the changes in mean physical component summary scores from the 2-month visit to the 4-month visit.

**Table 3:** Mean mental component summary scores and effect sizes by participant group and sex (N = 205)

	**Participants**	**Treated for tuberculosis disease**				**Treated for latent tuberculosis infection**				**Screened for tuberculosis but healthy and untreated**			
**VISIT**	**BY SEX**	**N (%)**	**Mean score (Standard Deviation)**	**Effect Size**_**1**_^**a**^	**Effect Size**_**2**_^**b**^	**N (%)**	**Mean score (Standard Deviation)**	**Effect Size**_**1**_^**a**^	**Effect Size**_**2**_^**b**^	**N (%)**	**Mean score (Standard Deviation)**	**Effect Size**_**1**_^**a**^	**Effect Size**_**2**_^**b**^
Baseline	Total	33	47.2 (6.5)	-	-	81	50.6 (4.7)	-	-	91	51.7 (4.3)	-	-
	Men	19 (58)	46.7 (7.6)	-	-	37 (46)	52.0 (3.2)	-	-	41 (45)	52.8 (3.5)	-	-
	Women	14 (42)	48.0 (4.9)	-	-	44 (54)	49.4 (5.4)	-	-	50 (55)	50.7 (4.8)	-	-
1 month	Total	36	50.9 (5.6)	**0.6**	-	58	50.1 (5.2)	−0.1	-	75	52.0 (4.3)	0.1	-
	Men	21 (58)	49.6 (5.4)	0.4	-	22 (38)	51.6 (3.9)	−0.1	-	38 (51)	52.0 (4.3)	−0.2	-
	Women	15 (42)	52.8 (5.5)	**0.9**	-	36 (62)	49.3 (5.6)	0.0	-	37 (49)	51.9 (4.3)	0.3	-
2 months	Total	39	52.2 (4.6)	**0.9**	0.3	52	51.0 (4.7)	0.1	0.2	77	52.1 (4.1)	0.1	0.0
	Men	23 (59)	51.0 (4.9)	**0.7**	0.3	23 (44)	52.4 (2.9)	0.1	0.2	37 (48)	52.6 (4.0)	−0.1	0.1
	Women	16 (41)	54.1 (3.5)	**1.4**	0.3	29 (56)	49.8 (5.5)	0.1	0.1	40 (52)	51.6 (4.3)	0.2	−0.1
4 months	Total	32	50.8 (6.6)	**0.5**	−0.3	53	51.1 (4.2)	0.1	0.0	56	52.1 (3.4)	0.1	0.0
	Men	19 (59)	49.1 (7.4)	0.3	−0.3	26 (49)	52.5 (3.6)	0.1	0.0	24 (43)	52.0 (3.6)	−0.2	−0.1
	Women	13 (41)	53.2 (4.4)	**1.1**	−0.2	27 (51)	49.8 (4.4)	0.1	0.0	32 (57)	52.1 (3.3)	0.3	0.1
6 months	Total	33	51.5 (5.8)	**0.7**	0.1	44	51.5 (3.9)	0.2	0.1	55	51.9 (4.1)	0.1	0.0
	Men	18 (55)	50.9 (5.9)	**0.6**	0.3	25 (57)	52.5 (2.6)	0.2	0.0	27 (49)	52.0 (3.8)	−0.2	0.0
	Women	15 (45)	52.2 (5.7)	**0.8**	−0.2	19 (43)	50.2 (5.0)	0.1	0.1	28 (51)	51.7 (4.5)	0.2	−0.1
9 months	Total	30	53.6 (3.5)	**1.2**	0.4	37	50.9 (4.8)	0.1	−0.1	44	52.0 (5.3)	0.1	0.0
	Men	18 (60)	53.2 (4.1)	**1.1**	**0.5**	20 (54)	51.6 (3.8)	−0.1	−0.3	18 (41)	53.4 (2.7)	0.2	0.4
	Women	12 (40)	54.0 (2.6)	**1.5**	0.4	17 (46)	50.2 (5.7)	0.1	0.0	26 (59)	50.9 (6.4)	0.0	−0.1
12 months	Total	21	54.3 (2.9)	**1.4**	0.2	42	51.3 (4.5)	0.1	0.1	49	52.9 (4.1)	0.3	0.2
	Men	13 (62)	53.9 (2.4)	**1.3**	0.2	21 (50)	51.2 (4.4)	−0.2	−0.1	23 (47)	53.2 (2.8)	0.1	−0.1
	Women	8 (38)	54.8 (3.7)	**1.6**	0.3	21 (50)	51.4 (4.7)	0.4	0.2	26 (53)	52.5 (5.0)	0.4	0.3

Effect size conveys the estimated magnitude in differences in two measures of scores. An effect size ≥0.50 indicates a statistically meaningful change in mean scores and is indicated in bold font in the table^15^.

^a^Effect Size_1_ is the effect size of the change in mean mental component summary scores from the baseline visit to the given visit.

^b^Effect size_2_ is the effect size of the change in mean mental component summary scores from the previous visit to the given visit, e.g. effect size_2_ for the 4-month visit is the effect size of the changes in mean mental component summary scores from the 2-month visit to the 4-month visit.

At baseline, mean domain scores reported by participants treated for TB disease were significantly lower than those reported by other participant groups except for mean mental health scores [Additional file 2]. Significantly lower mean physical functioning, role physical, and social functioning scores were reported at the 1-month visit by participants treated for TB disease. This participant group also reported significantly lower mean general health scores at the 2-month visit [Additional file 2].

Participants treated for TB disease reported improvement in mean domain scores from baseline to the 2-month visit (vitality and mental health), from baseline to the 4-month visit (role physical), or showed continuous improvement through the study period. Mean role physical, vitality, and mental health scores declined during 2 to 6 months of treatment, but improved again from the 6- to the 9-month visits [Additional file 2]. Statistical improvements were reported in (1) bodily pain and social functioning from baseline to each follow-up visit, (2) vitality from baseline to all follow-up visits except for the 6-month visit, (3) physical functioning, role physical, role emotional, and mental health from the baseline to the 2-, 4-, 6-, 9-, and 12-month visits, and (4) general health from the baseline to the 9- and 12-month visits [19] [Additional files 4,5,6,7,8,9,10,11].

Mean scores reported by participants treated for LTBI remained largely comparable with the control group [Figures 2, 3; Tables 2, 3; Additional files 2, 4, 5,6,7,8,9,10,11].

Participants treated for severe TB disease (26, 54%) reported lower mean scores compared to participants treated for non-severe TB disease (22, 46%) [Additional file 3].

### Findings from multivariable analyses of component summary scores

After adjustment, mean PCS scores reported by participants treated for TB disease did not change significantly throughout follow-up and were comparable to mean PCS scores reported by the control group [Table 4, Additional files 12,13].

**Table 4:** Mean physical component summary scores reported at each visit by participant group

**Visit**	**Crude estimate**	**Adjusted estimate** ^**a**^	**95% Confidence interval adjusted estimate**	P-value*
Baseline				
Tuberculosis disease	49.5	50.0	49.5 - 50.5	0.001*
Latent tuberculosis infection	50.3	50.6	50.3 - 51.0	0.37
Control^b^	50.2	50.7	50.1 - 51.2	-
1 month of treatment				
Tuberculosis disease	49.5	49.9	49.4 - 50.5	0.90
Latent tuberculosis infection	50.2	50.5	50.1 - 50.9	0.98
Control	50.2	50.7	50.1 - 51.3	-
2 months of treatment				
Tuberculosis disease	49.5	50.0	49.4 - 50.5	0.45
Latent tuberculosis infection	50.3	50.6	50.2 - 51.0	0.95
Control	50.2	50.8	50.3 - 51.3	-
4 months of treatment				
Tuberculosis disease	50.0	50.4	49.8 - 51.0	0.89
Latent tuberculosis infection	50.1	50.4	50.0 - 50.8	0.90
Control	50.1	50.8	50.2 - 51.5	-
6 months of treatment				
Tuberculosis disease	49.7	50.2	49.7 - 50.7	0.89
Latent tuberculosis infection	50.1	50.5	50.0 - 50.9	0.96
Control	50.1	50.8	50.3 - 51.4	-
9 months of treatment				
Tuberculosis disease	49.6	49.9	49.3 - 50.5	0.09
Latent tuberculosis infection	50.3	50.6	50.2 - 51.1	0.90
Control	50.1	51.1	50.5 - 51.8	-
12 months of treatment				
Tuberculosis disease	49.6	50.1	49.5 - 50.6	0.04*
Latent tuberculosis infection	50.2	50.6	50.1 - 51.0	0.50
Control	50.0	50.7	50.1 - 51.3	-

^a^Adjusted models comparing participants treated for tuberculosis disease to those participants in the untreated control group controlled for age at baseline, sex, other medication exposures at baseline (yes/no) reported in medical charts, and number of individuals residing in participants’ households. Adjusted models comparing participants treated for latent tuberculosis infection to those participants in the untreated control group controlled for age at baseline and sex.

^b^Control is a participant screened for tuberculosis who tested negative for tuberculosis and was found not to require treatment.

*Indicates a p-value less than 0.05 meaning a statistically significant difference in mean physical component summary scores reported by the group of treated participants and the participants in the control group in the adjusted model, at the given visit.

The adjusted mean MCS score reported by participants treated for TB disease was significantly lower than that reported by the control participants at the initial interview – mean MCS score reported by participants treated for TB disease was 46.4 (95% CI: 44.1, 48.8,); the control group reported a mean MCS score of 51.1 (95% CI: 48.9, 53.2) [Table 5]. Improvement in adjusted mean MCS scores was significantly greater among the participants with TB disease compared to the control participants, from the baseline to each follow-up visit and from the 6- to the 9-month visits [Additional files 14,15].

**Table 5:** Mean mental component summary scores reported at each visit by participant group

**Visit**	**Crude estimate**	**Adjusted estimate** ^**a**^	**95% Confidence interval adjusted estimate**	P-value*
Baseline				
Tuberculosis disease	47.1	46.4	44.1 - 48.8	<0.01*
Latent tuberculosis infection	50.5	50.5	48.5 - 52.4	0.07
Control^b^	51.7	51.1	48.9 - 53.2	-
1 month of treatment				
Tuberculosis disease	50.7	50.1	47.5 - 52.6	0.69
Latent tuberculosis infection	50.2	50.2	48.0 - 52.3	0.72
Control	51.5	50.8	48.6 - 53.1	-
2 months of treatment				
Tuberculosis disease	51.8	51.3	48.8 - 53.7	0.75
Latent tuberculosis infection	50.5	50.6	48.5 - 52.8	0.86
Control	51.7	51.2	49.2 - 53.2	-
4 months of treatment				
Tuberculosis disease	50.8	50.3	47.7 - 52.9	0.67
Latent tuberculosis infection	50.9	51.0	48.8 - 53.1	0.67
Control	51.9	51.4	49.1 - 53.8	-
6 months of treatment				
Tuberculosis disease	51.7	51.2	48.6 - 53.8	0.50
Latent tuberculosis infection	50.7	50.7	48.5 - 53.0	0.52
Control	51.5	51.0	48.7 - 53.4	-
9 months of treatment				
Tuberculosis disease	54.0	53.6	50.9 - 56.4	0.88
Latent tuberculosis infection	50.5	50.5	48.1 - 52.9	0.71
Control	51.4	51.0	48.5 - 53.5	-
12 months of treatment				
Tuberculosis disease	54.0	53.6	50.7 - 56.5	0.28
Latent tuberculosis infection	50.9	50.9	48.6 - 53.2	0.30
Control	52.2	51.7	49.3 - 54.2	-

^a^Adjusted models comparing participants treated for tuberculosis disease to those participants in the untreated control group controlled for age at baseline and sex. Adjusted models comparing participants treated for latent tuberculosis infection to those participants in the untreated control group controlled for age at baseline and sex.

^b^Control is a participant screened for tuberculosis who tested negative for tuberculosis and was found not to require treatment.

*Indicates a p-value less than 0.05 meaning a statistically significant difference in mean mental component summary scores reported by the group of treated participants and the participants in the control group in the adjusted model, at the given visit.

There were no statistically significant differences in adjusted mean scores or changes in adjusted scores reported by participants treated for LTBI or the control group [Tables 4,5; Additional files 12,13,14,15].

### Findings from sensitivity analyses

Results of linear mixed model regression including multiple imputation of missing data yielded similar results to those of the main models.

There were few differences in mean PCS and MCS scores among participants who attended, missed, and who were lost to follow-up; any differences appeared to be due to random variation [Additional files 16,17,18,19,20,21].

## Discussion

In this longitudinal study among a diverse population evaluated and/or treated for TB in Montreal, diagnosis and treatment of TB disease had a significant impact on HRQOL as measured by the SF-36, particularly with respect to its mental aspects. MCS scores improved throughout the study period, while PCS scores improved slightly from the 2- to the 4-month visits and declined again during the remainder of follow-up. At baseline, most domain scores among participants treated for TB disease were significantly lower than those among other participant groups. Domain scores reported by those treated for TB disease generally improved throughout follow-up.

Diagnosis and treatment for LTBI, on the other hand, had a minimal impact on HRQOL throughout the study period – mean scores reported by participants treated for LTBI were generally comparable to those reported by the control group.

Normative PCS and MCS scores for the Canadian population are 50.5 and 51.7, respectively [26]. Mean PCS scores reported by participants treated for TB disease in our study were similar to the Canadian normative PCS score. MCS scores among participants with TB disease were lower than Canadian values at baseline, but exceeded Canadian norms by the end of follow-up. Scores among participants treated for LTBI and control participants were similar to Canadian norms. The theory of social support proposes that support contributes to health by protecting people from the adverse effects of stress [27]. The majority of our study participants treated for TB disease were foreign-born persons who had arrived in Canada within the last 2–5 years. They may have experienced stresses related to immigration and/or social isolation in addition to those related to TB diagnosis and treatment. Providing additional support targeted at these stressors as part of TB care, especially at diagnosis and early in the treatment regimen, may improve HRQOL, particularly mental well-being.

The evolution of scores among participants treated for TB disease likely reflects both actual changes in physical and/or mental well-being over time, and response shift, *i.e.* changes in internal standards, values, and/or priorities [28]. Changes observed in mean MCS scores may be due to due to true change as well as response shift. Future analyses will examine the impact of potential response shift on reported HRQOL.

MCID thresholds are not known in the TB context, but are published for patients with other respiratory diseases [20.21]. Wyrwich *et. al.* (2005) calculated ranges of change across the eight domains of the SF-36 for patients treated for chronic obstructive pulmonary disease that indicated small change (8.3 – 12.5), moderate change (16.7 – 25), and large change (25 – 37.5). Corresponding figures for patients treated for asthma were 10 – 16.7, 20 – 33.3, and 30 – 50, respectively [21]. Mean improvements in domain scores reported by participants treated for TB disease, from the initial to the 12-month assessment, ranged from +10.3 points (general health) to +44.7 points (social functioning), suggesting small, moderate, and large changes across the domains of the SF-36.

Four other studies report PCS and MCS scores of patients treated for TB disease within two weeks of treatment initiation [2,11,29,30]. Mean component summary scores reported by our participants treated for TB disease were similar to those reported in our previous pilot study in Montreal (mean PCS score = 53, mean MCS score = 49), and to those described in a cohort treated for TB disease in Vancouver, Canada (mean PCS score = 48, mean MCS score = 43) [2,11]. A cohort treated for TB disease in London, the United Kingdom reported lower mean PCS (36) and MCS (42) scores, while a group treated for TB disease in Uganda reported higher scores; mean PCS and MCS scores were 61 [29,30].

Similar to our findings, Kruijshaar *et. al.* reported improvement in mean MCS but not mean PCS scores from diagnosis to two months of treatment among patients treated for TB disease in London, the United Kingdom [1,29]. However, Marra *et. al.* did not find any significant improvement in mean PCS or MCS scores from diagnosis to six months of treatment among participants treated for TB disease in Vancouver [1,2].

Many factors may contribute to the similarities and differences observed between our findings and the results reported by other studies. It is not surprising that results in two Canadian cities (Montreal and Vancouver) were generally comparable. However, although most persons treated for TB in London and in Canadian cities are immigrants, their demographics, views, and experiences may differ. HRQOL and TB are interpreted differently across cultures and settings, which may also explain differences with the results from the Ugandan study.

As expected, participants in our study treated for more severe TB disease reported somewhat poorer HRQOL compared to those treated for non-severe TB disease. However, this analysis should be interpreted cautiously given the small size of these sub-groups.

Our study is the first to concurrently evaluate HRQOL of individuals treated for TB disease, LTBI, and an untreated comparison group at each milestone of treatment. The control group, with a similar demographic profile to the other participant groups, helps tease apart the impact of diagnosis and treatment for TB disease and LTBI from other stressors, most notably those related to immigration. As such, decrements in HRQOL reported by participants treated for TB disease are likely related to the diagnosis and treatment of this disease rather than to external stressors. The fact that participants treated for severe TB disease reported the poorest mean scores for physical health domains is consistent with this premise.

There are several limitations of our study, beginning with our inability to document HRQOL while persons were ill with TB disease, but not yet diagnosed. This would tend to underestimate the impact of symptomatic TB disease on HRQOL. Previous studies of symptomatic patients diagnosed with TB disease estimate that on average, such individuals experience symptoms for three months before diagnosis; the interval is substantially longer in resource-limited settings [31,32].

Selection bias may also have affected our estimates of HRQOL. First, participation in this study required English or French language skills. Second, we had very little data to characterize individuals who refused to participate. Third, participants who missed visits or who were lost to follow-up are of particular concern among participants treated for LTBI, as those who experienced disruptive treatment side effects may have stopped their medication and follow-up visits. However, our sensitivity analyses indicated comparable mean PCS and MCS scores among participants who subsequently attended visits, missed visits, or who were lost to follow-up.

Our study was conducted in a low TB disease incidence setting with an overwhelming majority of immigrants. Some high-risk groups (immigrants without English or French language skills, and Aboriginal peoples) were not well-represented in this study. Our results, therefore, may not be generalizable to high-incidence, resource-limited settings and to groups underrepresented in this study.

Finally, our analysis was limited in that we did not use a mixed methods approach; we did not collect qualitative data to expand on the findings of this quantitative analysis. Recent findings from qualitative interviews of patients treated for TB disease suggest a similar negative impact of TB disease and diagnosis, particularly on mental well-being, as we found in our quantitative analysis [33-35]. Future research should consider capturing patient perspectives using both quantitative and qualitative approaches.

## Conclusion

We observed a significant negative impact of TB disease on HRQOL in a diverse sample of patients treated in Montreal. While all domains were affected, the greatest impact was on mental health dimensions, which improved throughout the study period. The diagnosis and treatment of LTBI had little effect on HRQOL.

Our results highlight impaired mental well-being but unaltered physical well-being of participants treated for TB disease, particularly in the early months of treatment. These findings suggest a potential role for targeted, culturally relevant psychosocial support interventions for persons treated for TB disease, especially during the early months of treatment. For example, participants treated for TB disease reported particular decrements in social functioning early in treatment [Additional file 2]. Based on this finding, health care policy makers may want to focus on ending respiratory isolation as soon as possible and offer supportive services that integrate patients back into their communities as quickly as possible. In this way, our results may be used to guide health care professionals and health care policy makers in designing new approaches to patient care that address gaps in the well-being of persons treated for TB disease in Canada.

## Additional files

Additional file 1: Table S1. Clinical characteristics at the initial interview by participant group and sex. Data describes diagnostic test results, measures of disease severity, treatment regimens, and treatment administration, stratified by participant group and sex. Additional file 2: Figure S1. Mean domain scores and 95% confidence intervals reported at each visit by participant group. Mean scores and 95% confidence intervals of each of the eight domains of the SF-36 questionnaire as reported by each participant group at each visit. Additional file 3: Figure S2. Mean SF-36 summary component scores and domain scores and 95% confidence intervals reported at each visit by participants treated for tuberculosis disease, stratified by disease severity. Mean scores and 95% confidence intervals of the two summary component scores and eight domains of the SF-36 questionnaire as reported at each visit by (1) participants treated for severe TB disease, and (2) participants treated for non-severe TB disease. Additional file 4: Table S4. Mean physical functioning scores and effect sizes at each follow-up visit by participant group and sex (N = 258). Mean SF-36 physical functioning scores and standard deviations reported at each visit, stratified by participant group and sex. Two effect size measures were calculated for each stratum: (1) the effect size between mean scores from baseline to each follow-up visit, and (2) the effect size between mean scores from a given visit to the next consecutive visit. Additional file 5: Figure S5. Mean role physical scores and effect sizes at each follow-up visit by participant group and sex (N = 258). Mean SF-36 role physical scores and standard deviations reported at each visit, stratified by participant group and sex. Two effect size measures were calculated for each stratum: (1) the effect size between mean scores from baseline to each follow-up visit, and (2) the effect size between mean scores from a given visit to the next consecutive visit. Additional file 6: Table S6. Mean bodily pain scores and effect sizes at each follow-up visit by participant group and sex (N = 219). Mean SF-36 bodily pain scores and standard deviations reported at each visit, stratified by participant group and sex. Two effect size measures were calculated for each stratum: (1) the effect size between mean scores from baseline to each follow-up visit, and (2) the effect size between mean scores from a given visit to the next consecutive visit. Additional file 7: Table S7. Mean general health scores and effect sizes at each follow-up visit by participant group and sex (N = 258). Mean SF-36 general health scores and standard deviations reported at each visit, stratified by participant group and sex. Two effect size measures were calculated for each stratum: (1) the effect size between mean scores from baseline to each follow-up visit, and (2) the effect size between mean scores from a given visit to the next consecutive visit. Additional file 8: Table S8. Mean vitality scores and effect sizes at each follow-up visit by participant group and sex (N = 253). Mean SF-36 vitality scores and standard deviations reported at each visit, stratified by participant group and sex. Two effect size measures were calculated for each stratum: (1) the effect size between mean scores from baseline to each follow-up visit, and (2) the effect size between mean scores from a given visit to the next consecutive visit. Additional file 9: Table S9. Mean social functioning scores and effect sizes at each follow-up visit by participant group and sex (N = 258). Mean SF-36 social functioning scores and standard deviations reported at each visit, stratified by participant group and sex. Two effect size measures were calculated for each stratum: (1) the effect size between mean scores from baseline to each follow-up visit, and (2) the effect size between mean scores from a given visit to the next consecutive visit. Additional file 10: Table S10. Mean role emotional scores and effect sizes at each follow-up visit by participant group and sex (N = 258). Mean SF-36 role emotional scores and standard deviations reported at each visit, stratified by participant group and sex. Two effect size measures were calculated for each stratum: (1) the effect size between mean scores from baseline to each follow-up visit, and (2) the effect size between mean scores from a given visit to the next consecutive visit. Additional file 11: Table S11. Mean mental health scores and effect sizes at each follow-up visit by participant group and sex (N = 256). Mean SF-36 mental health scores and standard deviations reported at each visit, stratified by participant group and sex. Two effect size measures were calculated for each stratum: (1) the effect size between mean scores from baseline to each follow-up visit, and (2) the effect size between mean scores from a given visit to the next consecutive visit. Additional file 12: Table S12. Changes in mean physical component summary scores reported since the baseline visit to each follow-up visit by each participant group. Using linear mixed models and controlling for important confounders, these data present changes in mean SF-36 physical component summary scores (both crude and adjusted estimates) from baseline to each follow-up visit. The 95% confidence intervals of adjusted estimates are presented in this file, along with p-values indicating the statistical significance of the difference in adjusted estimates between each of the treated participant groups and the control group at each time interval. Additional file 13: Table S13. Changes in mean physical component summary scores reported at each follow-up interval by each participant group. Using linear mixed models and controlling for important confounders, these data present changes in mean SF-36 physical component summary scores (both crude and adjusted estimates) from a given visit to each consecutive follow-up visit. The 95% confidence intervals of adjusted estimates are presented in this file, along with p-values indicating the statistical significance of the difference in adjusted estimates between each of the treated participant groups and the control group at each time interval. Additional file 14: Table S14. Changes in mean mental component summary scores reported since the baseline visit to each follow-up visit by each participant group. Using linear mixed models and controlling for important confounders, these data present changes in mean SF-36 mental component summary scores (both crude and adjusted estimates) from baseline to each follow-up visit. The 95% confidence intervals of adjusted estimates are presented in this file, along with p-values indicating the statistical significance of the difference in adjusted estimates between each of the treated participant groups and the control group at each time interval. Additional file 15: Table S15. Changes in mean mental component summary scores reported at each follow-up interval by each participant group. Using linear mixed models and controlling for important confounders, these data present changes in mean SF-36 mental component summary scores (both crude and adjusted estimates) from a given visit to each consecutive follow-up visit. The 95% confidence intervals of adjusted estimates are presented in this file, along with p-values indicating the statistical significance of the difference in adjusted estimates between each of the treated participant groups and the control group at each time interval. Additional file 16: Table S16. Adjusted mean SF-36 physical component summary scores calculated using multiple imputation, reported at each follow-up visit by participant group. Using linear mixed models and controlling for important confounders, these data present adjusted estimates of mean SF-36 physical component summary scores (calculated using multiple imputation) reported at each visit for each participant group. The 95% confidence intervals of the adjusted estimates are also shown, along with p-values indicating the statistical significance of the difference in adjusted estimates between each of the treated participant groups and the control group at each visit. Additional file 17: Table S17. Adjusted changes in mean SF-36 physical component summary scores calculated using multiple imputation reported since the baseline visit to each follow-up interval, by each participant group. Using linear mixed models and controlling for important confounders, these data present adjusted estimates of changes in mean SF-36 physical component summary scores (calculated using multiple imputation) reported from the baseline visit to each follow-up visit, for each participant group. The 95% confidence intervals of the adjusted estimates are also shown, along with p-values indicating the statistical significance of the difference in adjusted estimates between each of the treated participant groups and the control group at each time interval. Additional file 18: Table S18. Adjusted changes in mean SF-36 physical component summary scores calculated using multiple imputation reported at each follow-up interval, by participant group. Using linear mixed models and controlling for important confounders, these data present adjusted estimates of changes in mean SF-36 mental component summary scores (calculated using multiple imputation) reported from a given visit to each consecutive follow-up visit, for each participant group. The 95% confidence intervals of the adjusted estimates are also shown, along with p-values indicating the statistical significance of the difference in adjusted estimates between each of the treated participant groups and the control group at each time interval. Additional file 19: Table S19. Adjusted mean SF-36 mental component summary scores calculated using multiple imputation, reported at each follow-up visit by participant group. Using linear mixed models and controlling for important confounders, these data present adjusted estimates of mean SF-36 mental component summary scores (calculated using multiple imputation) reported at each visit for each participant group. The 95% confidence intervals of the adjusted estimates are also shown, along with p-values indicating the statistical significance of the difference in adjusted estimates between each of the treated participant groups and the control group at each visit. Additional file 20: Table S20. Adjusted changes in mean SF-36 mental component summary scores calculated using multiple imputation reported since the baseline visit to each follow-up interval, by each participant group. Using linear mixed models and controlling for important confounders, these data present adjusted estimates of changes in mean SF-36 mental component summary scores (calculated using multiple imputation) reported from the baseline visit to each follow-up visit, for each participant group. The 95% confidence intervals of the adjusted estimates are also shown, along with p-values indicating the statistical significance of the difference in adjusted estimates between each of the treated participant groups and the control group at each time interval. Additional file 21: Table S21. Adjusted changes in mean SF-36 mental component summary scores calculated using multiple imputation reported at each follow-up interval, by participant group. Using linear mixed models and controlling for important confounders, these data present adjusted estimates of changes in mean SF-36 mental component summary scores (calculated using multiple imputation) reported from a given visit to each consecutive follow-up visit, for each participant group. The 95% confidence intervals of the adjusted estimates are also shown, along with p-values indicating the statistical significance of the difference in adjusted estimates between each of the treated participant groups and the control group at each time interval.

## Abbreviations

TB: Tuberculosis
LTBI: Latent tuberculosis infection
HRQOL: Health-related quality of life
SF-36: Short Form-36 questionnaire version 2
PCS: Physical Component Summary (score) of the SF-36
MCS: Mental Component Summary (score) of the SF-36
INH: Isoniazid
MCI: Montreal Chest Institute
JGH: Sir Mortimer B. Davis Jewish General Hospital
TST: Tuberculin Skin Test
IGRA: Interferon-γ Release Assay
MCID: Minimal clinically important difference
AIC: Akaike Information Criterion
MI: Multiple imputation
