# Supplementary figures and images for: Health-related quality of life and tuberculosis: a longitudinal cohort study
Source: Health Qual Life Outcomes. 2015 May 27;13:65. doi: 10.1186/s12955-015-0250-4 (PMC4443601; doi:10.1186/s12955-015-0250-4)

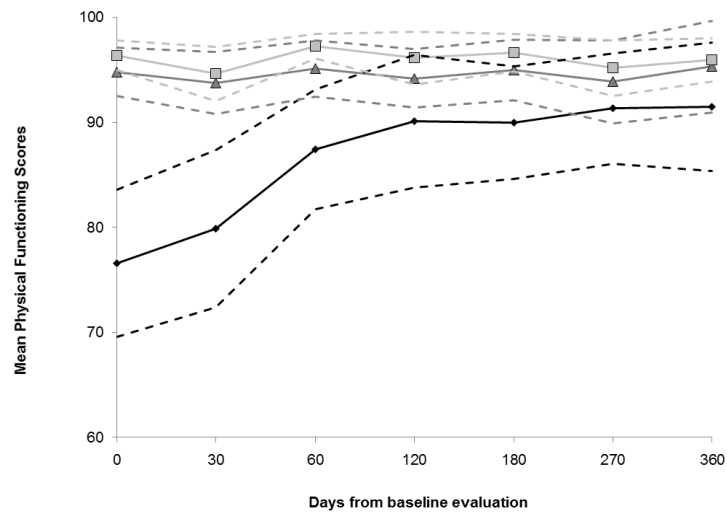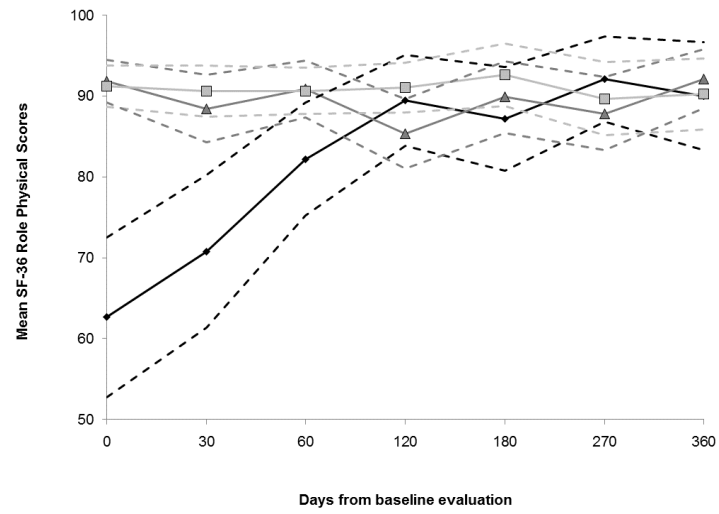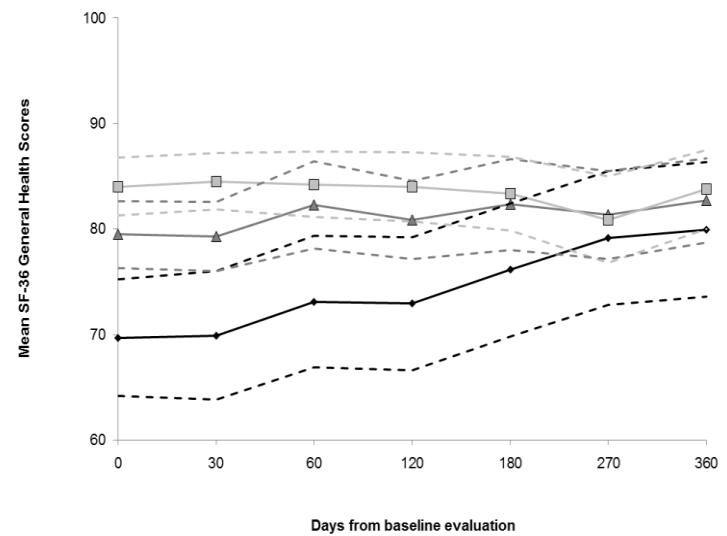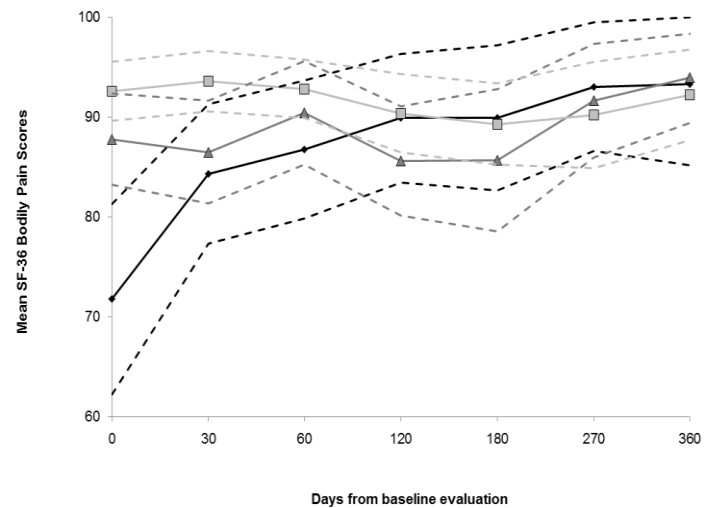

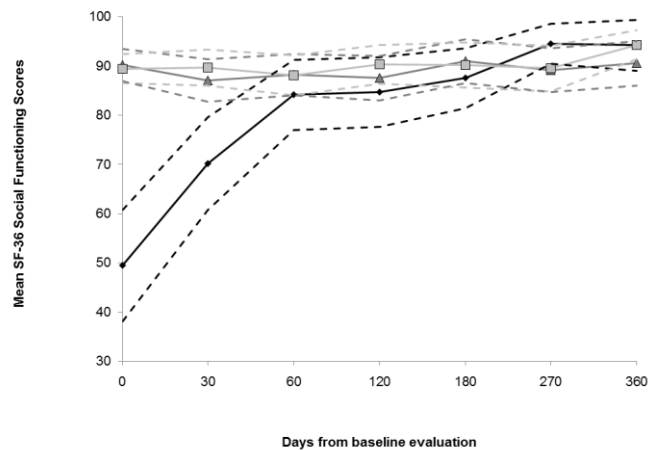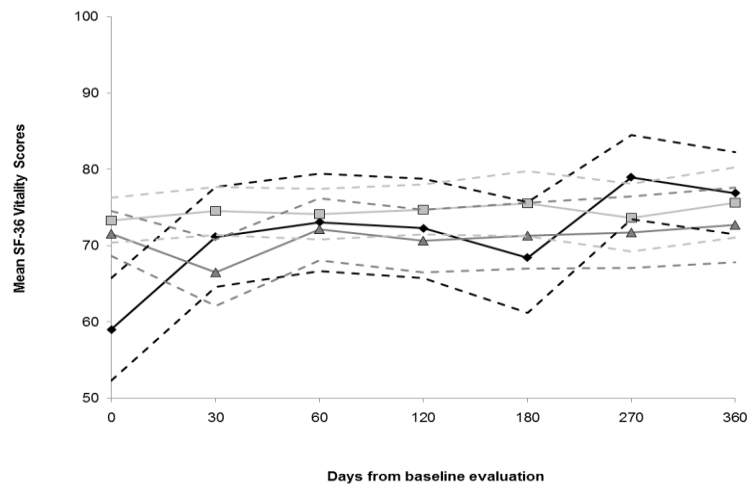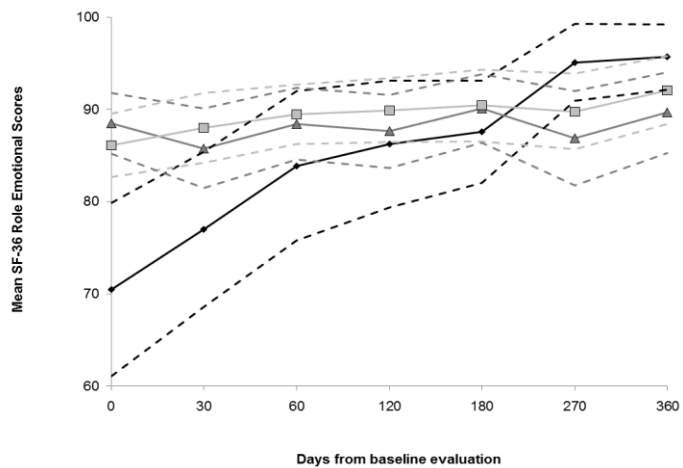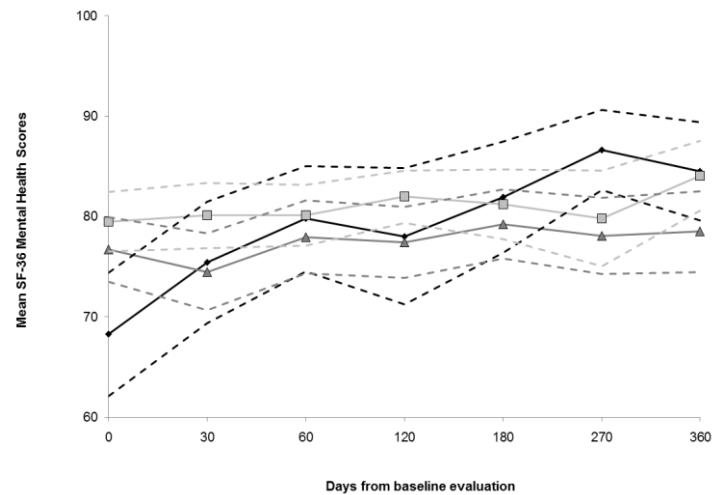

Supplement: Additional file 2: Figure S1. — Mean domain scores and 95% confidence intervals reported at each visit by participant group. Mean scores and 95% confidence intervals of each of the eight domains of the SF-36 questionnaire as reported by each participant group at each visit. [file 12955_2015_250_MOESM2_ESM.pdf]

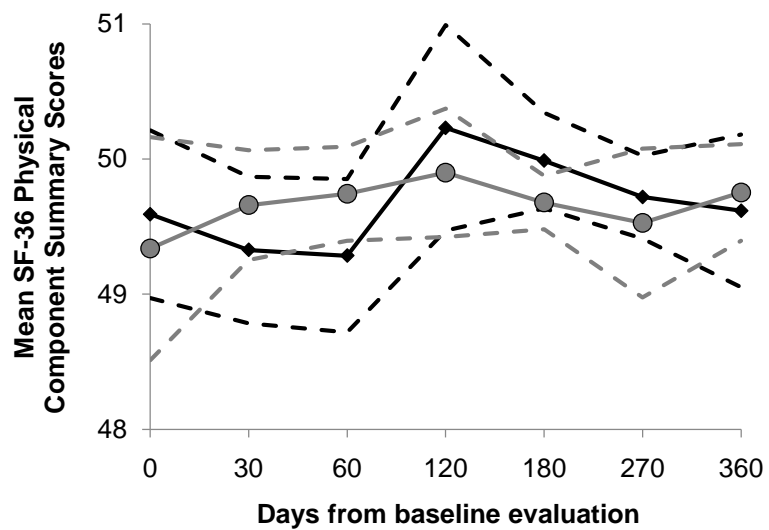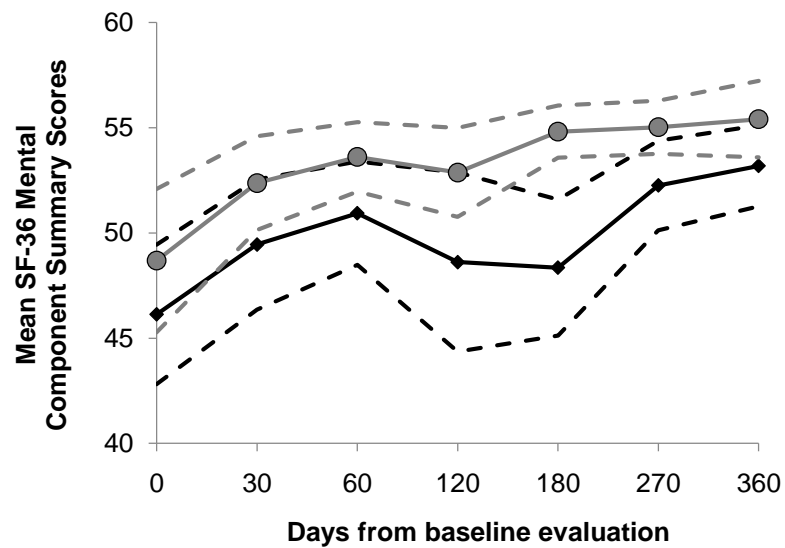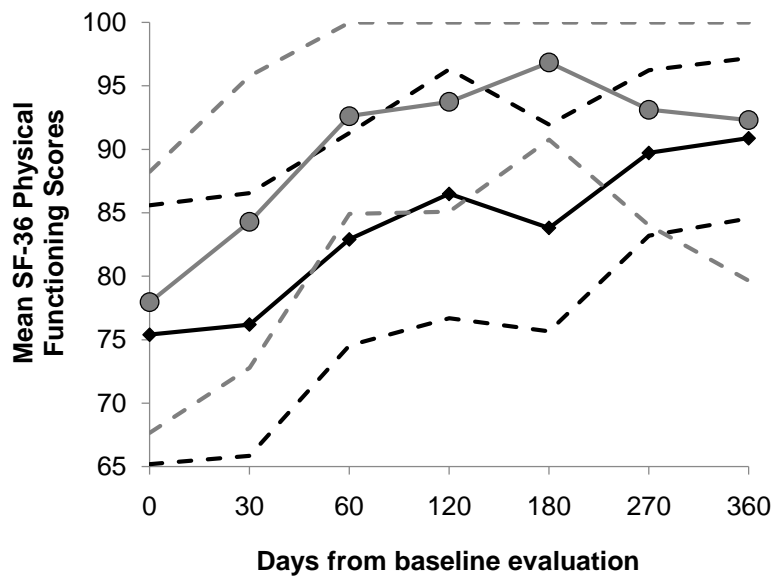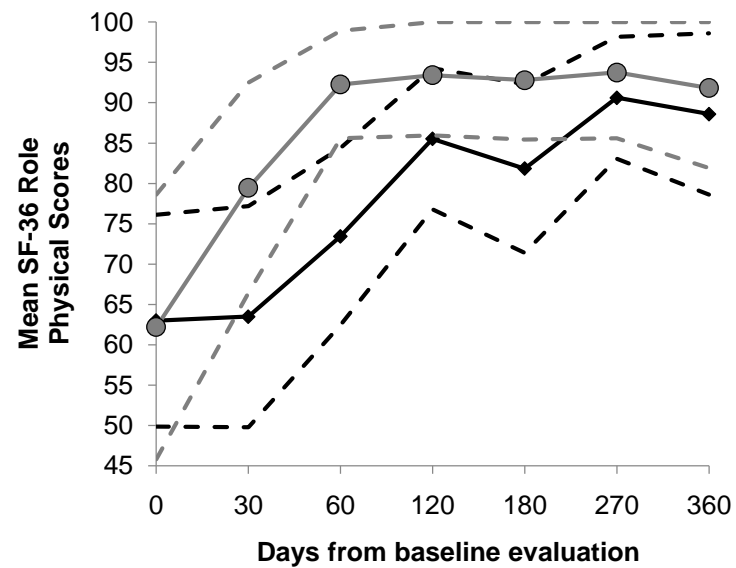

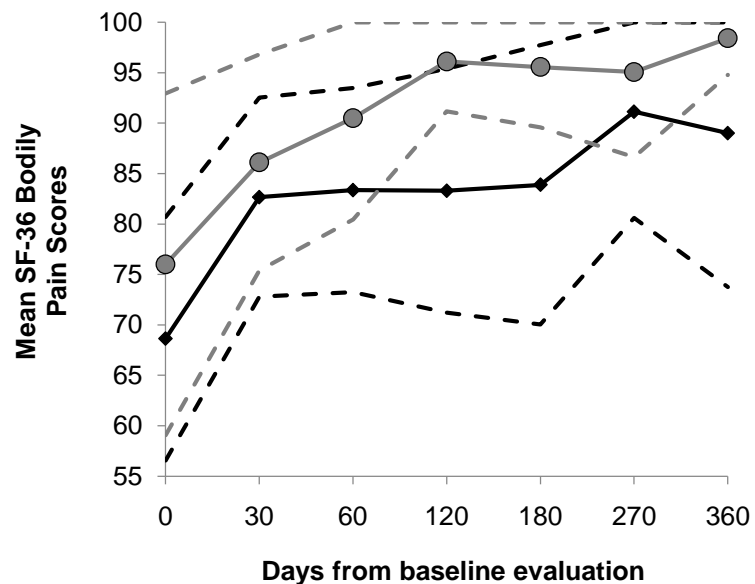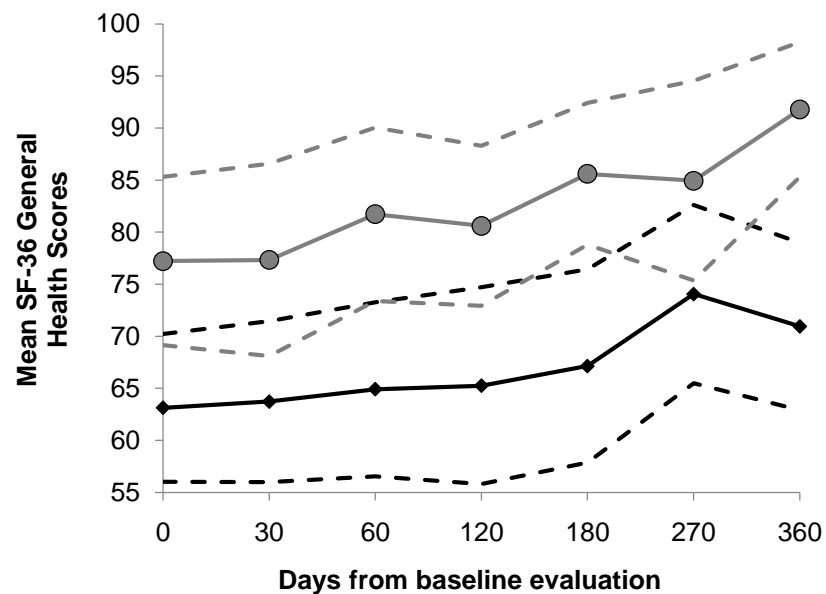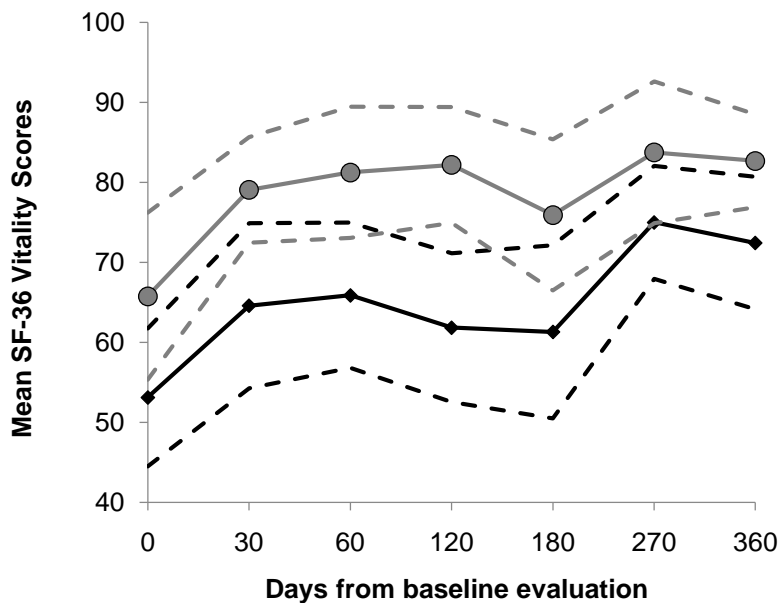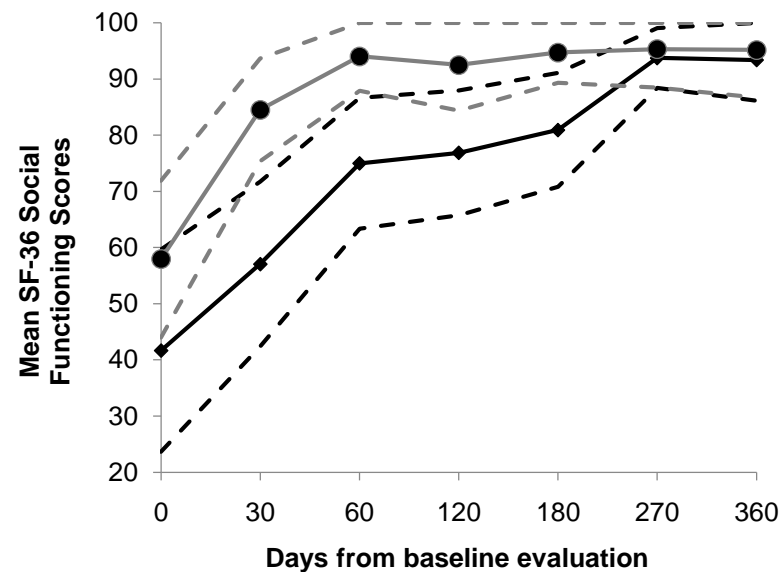

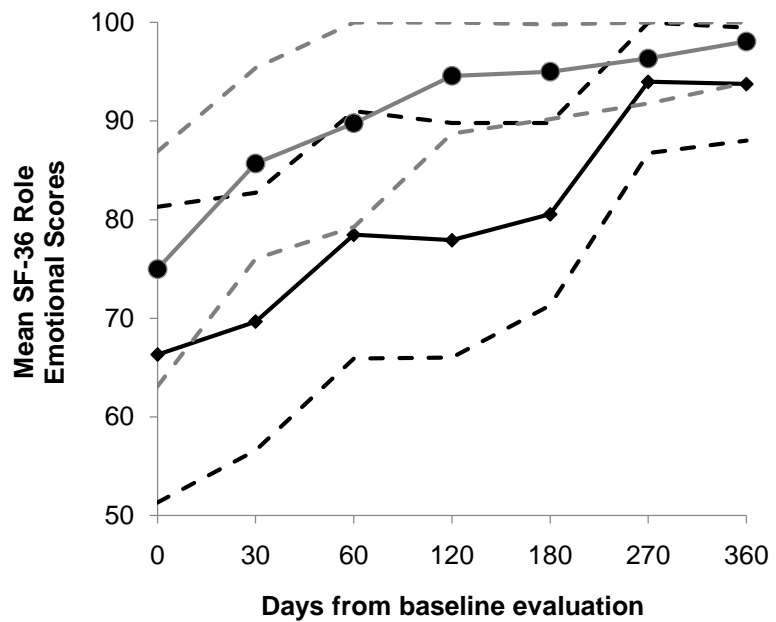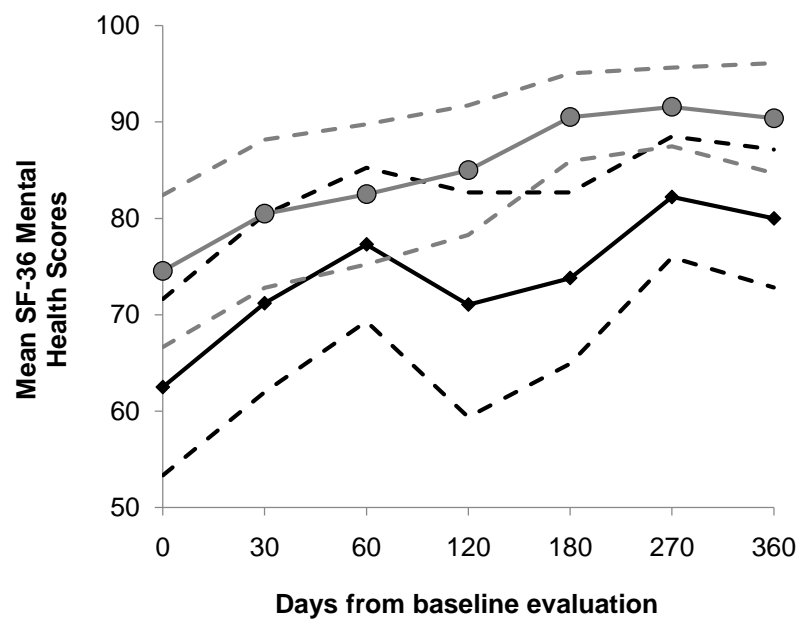

Supplement: Additional file 3: Figure S2. — Mean SF-36 summary component scores and domain scores and 95% confidence intervals reported at each visit by participants treated for tuberculosis disease, stratified by disease severity. Mean scores and 95% confidence intervals of the two summary component scores and eight domains of the SF-36 questionnaire as reported at each visit by (1) participants treated for severe TB disease, and (2) participants treated for non-severe TB disease. [file 12955_2015_250_MOESM3_ESM.pdf]
